# Supplementary material for: Analysis of single-cell transcriptomes links enrichment of olfactory receptors with cancer cell differentiation status and prognosis
Source: Commun Biol. 2020 Sep 11;3:506. doi: 10.1038/s42003-020-01232-5 (PMC7486295; doi:10.1038/s42003-020-01232-5)
Supplement: Supplementary file 1 — Description of Additional Supplementary Files [file 42003_2020_1232_MOESM1_ESM.pdf]

SUPPLEMENTARY DATA

Supplementary Data 1: The table contains information about the datasets used in this study. It also contains information about the number and identity of the expressed chemoreceptors (functional ORs, pseudo-ORs, V1Rs, T1Rs, T2Rs, and TAARs) identified in these datasets.

Supplementary Data 2: The table contains information about the cancer-associated ORs and their source (tumor or cell lines).

Supplementary Data 3: The table contains information about the functional relevance of the cancer-associated ORs. It contains the correlation values for the OR expression and Gene set variation analysis (GSVA) scores of the indicated tumor-related signatures.

Supplementary Data 4: The table contains a list of all the olfactory receptors identified in the healthy breast epithelial cells, malignant epithelial cells derived from patient-derived xenograft, breast carcinoma tissue, and circulating breast epithelial cells.

Supplementary Data 5: The table contains information about the breast carcinoma-associated ORs along with the information about molecular subtypes of the malignant epithelial cells, ORs median expression values, and their statistical inference.

Supplementary Data 6: The table contains information about the upregulated and downregulated genes in different clusters of BRCA.

Supplementary Data 7: The table contains information about the parameters used for pseudo temporal analysis by Monocle on breast carcinoma and healthy breast epithelial datasets.

Supplementary Data 8: The table contains information about the tumor stage of the TCGA patient in five subgroups, segregated based on OR-centric signatures.
