# Supplementary material for: Analysis of single-cell transcriptomes links enrichment of olfactory receptors with cancer cell differentiation status and prognosis
Source: Commun Biol. 2020 Sep 11;3:506. doi: 10.1038/s42003-020-01232-5 (PMC7486295; doi:10.1038/s42003-020-01232-5)
Supplement: Supplementary file 2 — Supplementary Information [file 42003_2020_1232_MOESM2_ESM.pdf]

## Supplementary Figure Legends

**Supplementary Figure 1: CancerSmell: A computational workflow to determine the expression status of chemosensory receptors from single-cell datasets.**

**a** Schematic representation of the computational workflow (Cancer Smell) to systematically estimate the activation status of the chemosensory receptors at the single-cell resolution.

**b** Graphical illustration of the scRNA-seq datasets (tumor and cell lines) used in the study.

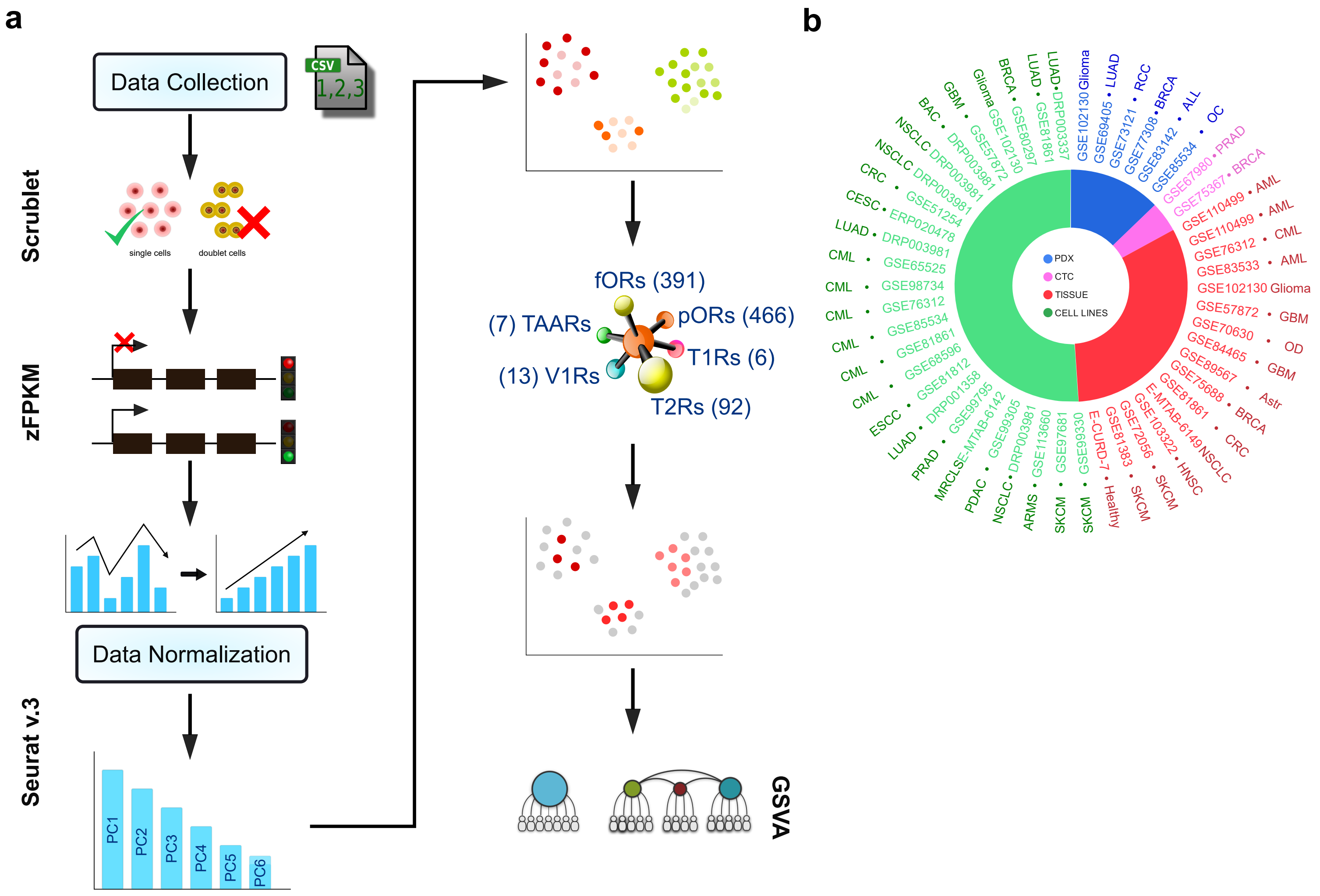


**Supplementary Figure 1**

**Supplementary Figure 2: Comprehensive map of tumor/cell lines-associated ORs.**

**a** Chord diagram summarizing the relationship between all identified tumor-associated ectopic ORs and the tumor-types.

**b** Chord diagram summarizing the relationship between all identified tumor-associated ectopic ORs and the cancer cell lines.

**c** Bar graph representing the percentage of OR-positive malignant cells in the indicated cancer cell lines. zFPKM algorithm was used for the determination of the reliably expressed ORs (zFPKM > -3, activated).

**d** Uniform Manifold Approximation and Projection (UMAP) depicting the relative expression of the taste receptor (TAS1R3) in the indicated single-cell cell line dataset.

**e** Uniform Manifold Approximation and Projection (UMAP) depicting the relative expression of the vomeronasal receptor (VN1R1) in the indicated single-cell cell line dataset.

**f** Uniform Manifold Approximation and Projection (UMAP) depicting the relative expression of the vomeronasal receptor (VN1R2) in the indicated single-cell cell line dataset.

**g** Box plot representing the correlation between GSVA scores for the indicated signatures and

the expression of the cancer-associated ORs. The color of the box represents distinct tumor signatures.

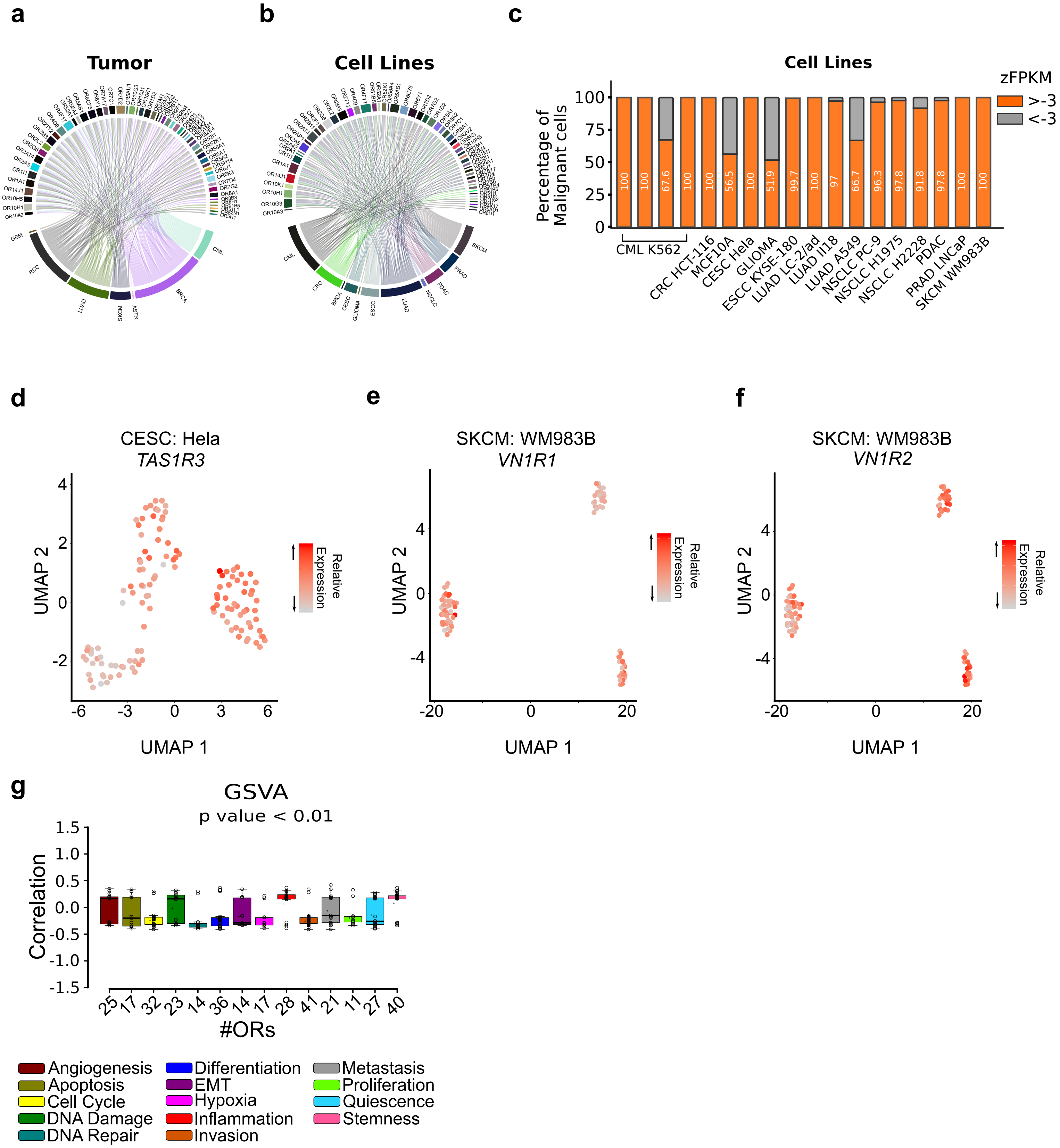


**Supplementary Figure 2**

**Supplementary Figure 3: Cellular count of expressed ORs largely varies across multiple cancer cell lines.**

**a** Percentage bar graph depicting the cellular count of expressed ORs in the indicated tumor cell lines. zFPKM algorithm was used for the determination of the OR activation status (zFPKM > -3, activated).

**b** Uniform Manifold Approximation and Projection (UMAP) depicting the distinct cellular clusters segregated based on their transcriptomes. The molecular identity of different clusters is also labeled.

**c** Bar graph representing the number of cells in different molecular subtypes of breast carcinoma.

**d** Correlation plot depicting the transcriptomic similarities between pooled and respective single malignant cells.

**e** Whisker plot representing the number of reliably detected ORs in the indicated conditions.

**f** Whisker plot representing the median expression of the breast carcinoma-associated ORs in the indicated conditions.

**g** Density-histograms depicting the normalized expression of the indicated ORs in the single-cell breast carcinoma dataset.

**h** Correlation plot depicting the expression similarities between breast carcinoma-associated ORs.

**i** Metascape plot depicting the cluster information of the single-cell breast carcinoma dataset.

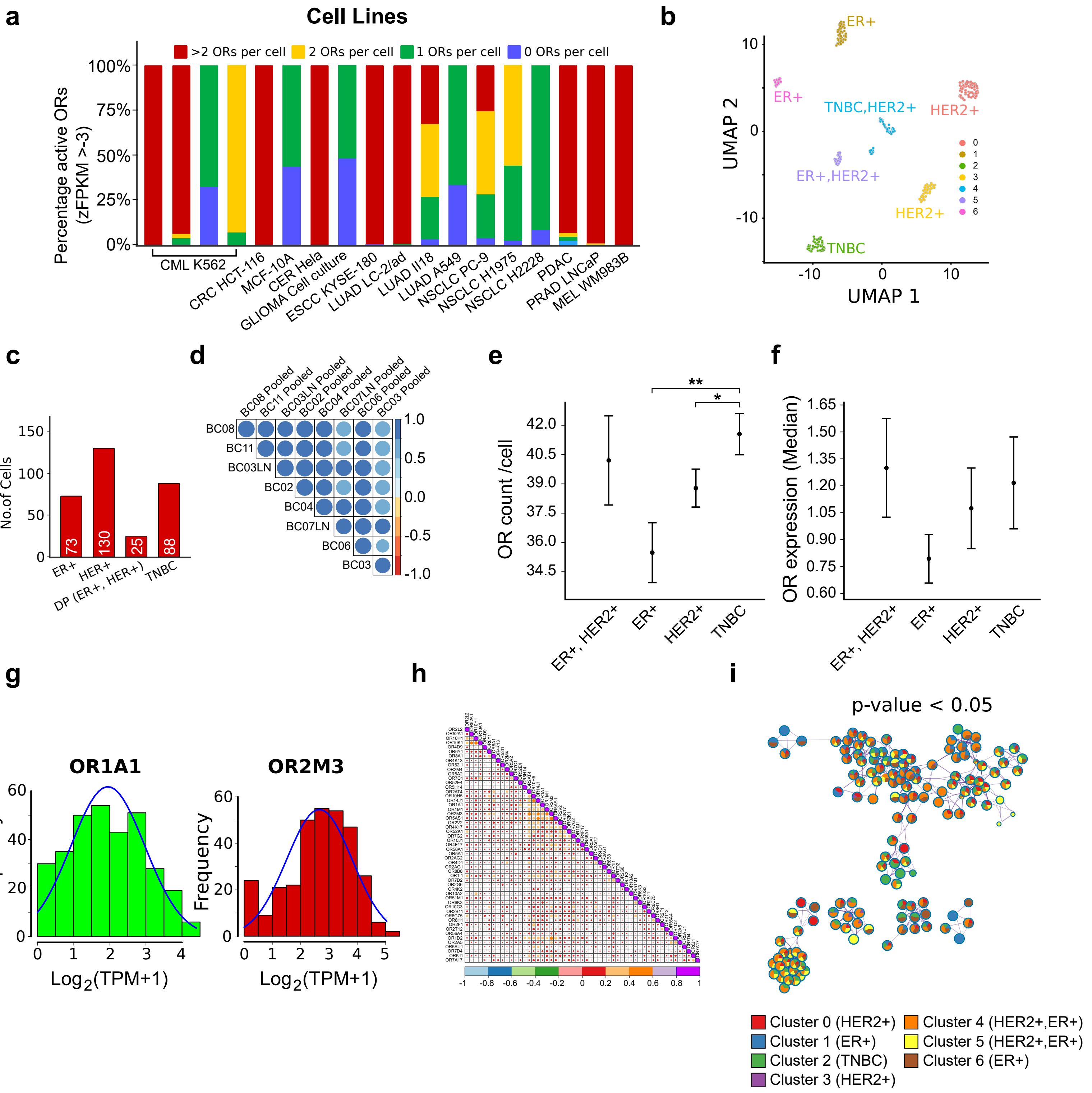


**Supplementary Figure 3**

**Supplementary Figure 4: Differentiation-dependent decline in the cellular count of the expressed ORs is specific to malignant cells.**

**a** Monocle-generated pseudotemporal trajectory of malignant breast epithelial cells. Arrowheads indicating the direction of the cellular differentiation.

**b** Uniform Manifold Approximation and Projection (UMAP) plot representing the number of active OR genes per malignant cell. Arrowheads indicate the direction of cellular differentiation.

**c** Uniform Manifold Approximation and Projection (UMAP) plot depicting the relative cellular stemness across all malignant breast carcinoma cells.

**d** Uniform Manifold Approximation and Projection (UMAP) plot depicting the tumor stages of the cells across the pseudotemporal trajectory.

**e** Scatter plot depicting the correlation between cellular stemness and cell differentiation time-points (pseudotime).

**f** Scatter plot depicting the correlation between mean expression of the breast carcinoma-associated ORs and cell differentiation time-points (pseudotime).

**g** Scatter plot depicting the correlation between the cellular count of expressed ORs in the breast carcinoma and cancer cell differentiation time-points (pseudotime).

**h** Scatter plot depicting the negative correlation between cellular stemness and cell differentiation time-points (pseudotime).

**i** Scatter plot depicting the negative correlation between the cellular count of expressed ORs in breast carcinoma and cancer cell differentiation time-points (pseudotime).

**j** Scatter plot depicting the positive correlation between the cellular count of expressed ORs in breast carcinoma and cellular stemness.

**k** Uniform Manifold Approximation and Projection (UMAP) depicting cellular stemness along the pseudotemporal trajectory in the healthy luminal breast epithelial cells.

**l** Scatter plot depicting the correlation between cellular stemness and cell differentiation time-points (pseudotime) in the healthy breast epithelial dataset.

**m** Scatter plot depicting the correlation between the cellular count of expressed ORs in the healthy breast epithelial dataset and cellular differentiation time-points (pseudotime).

**n** Scatter plot depicting the correlation between the cellular count of expressed ORs in the healthy breast epithelial dataset and cellular stemness.

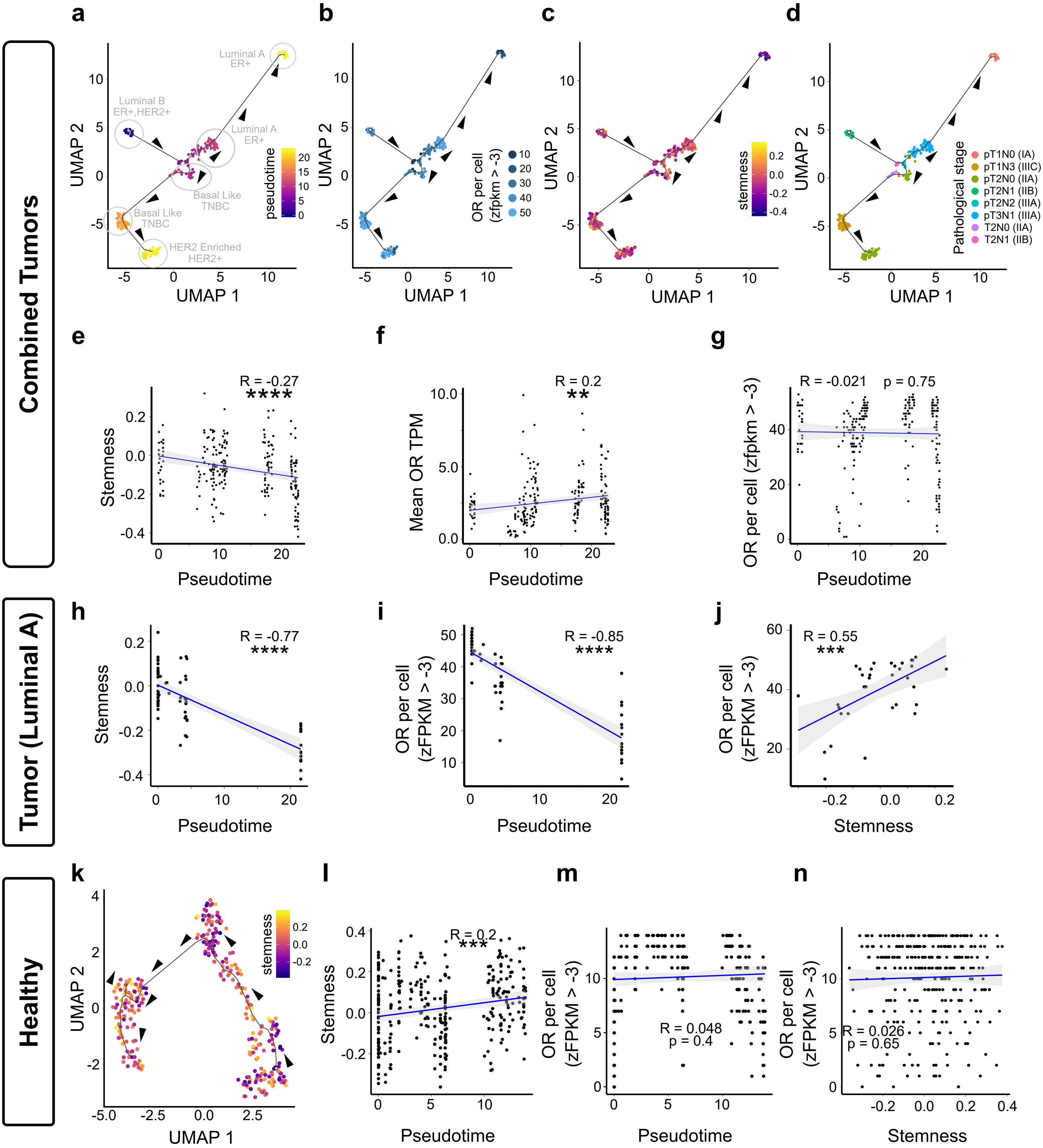


Supplementary Figure 4

**Supplementary Figure 5: Sub-classification of bulk tumor profiles using single-cell derived OR-centric signatures**

**a** Heatmap, along with the cellular clustering (columns) depicting the segregation of cells based on OR expression status in breast carcinoma (tissue-derived single-cell dataset).

**b** Heatmap, along with the cellular clustering (columns) depicting the segregation of cells based on OR expression status in breast carcinoma (single-cell dataset of circulating tumor cells of breast carcinoma).

**c** Heatmap, along with the cellular clustering (columns) depicting the segregation of cells based on OR expression status in breast carcinoma (single-cell dataset of patient-derived xenografts of breast carcinoma).

**d** Heatmap depicting the cosine similarity between the breast cancer (tissue) scRNA-sequencing derived OR-centric signatures and stratified TCGA patient cohorts.

**e** Heatmap depicting the cosine similarity between the breast cancer (circulating tumor cells) scRNA-sequencing derived OR-centric signatures and stratified TCGA patient cohorts.

**f** Heatmap depicting the cosine similarity between the breast cancer (patient-derived xenografts) scRNA-sequencing derived OR-centric signatures and stratified TCGA patient cohorts.

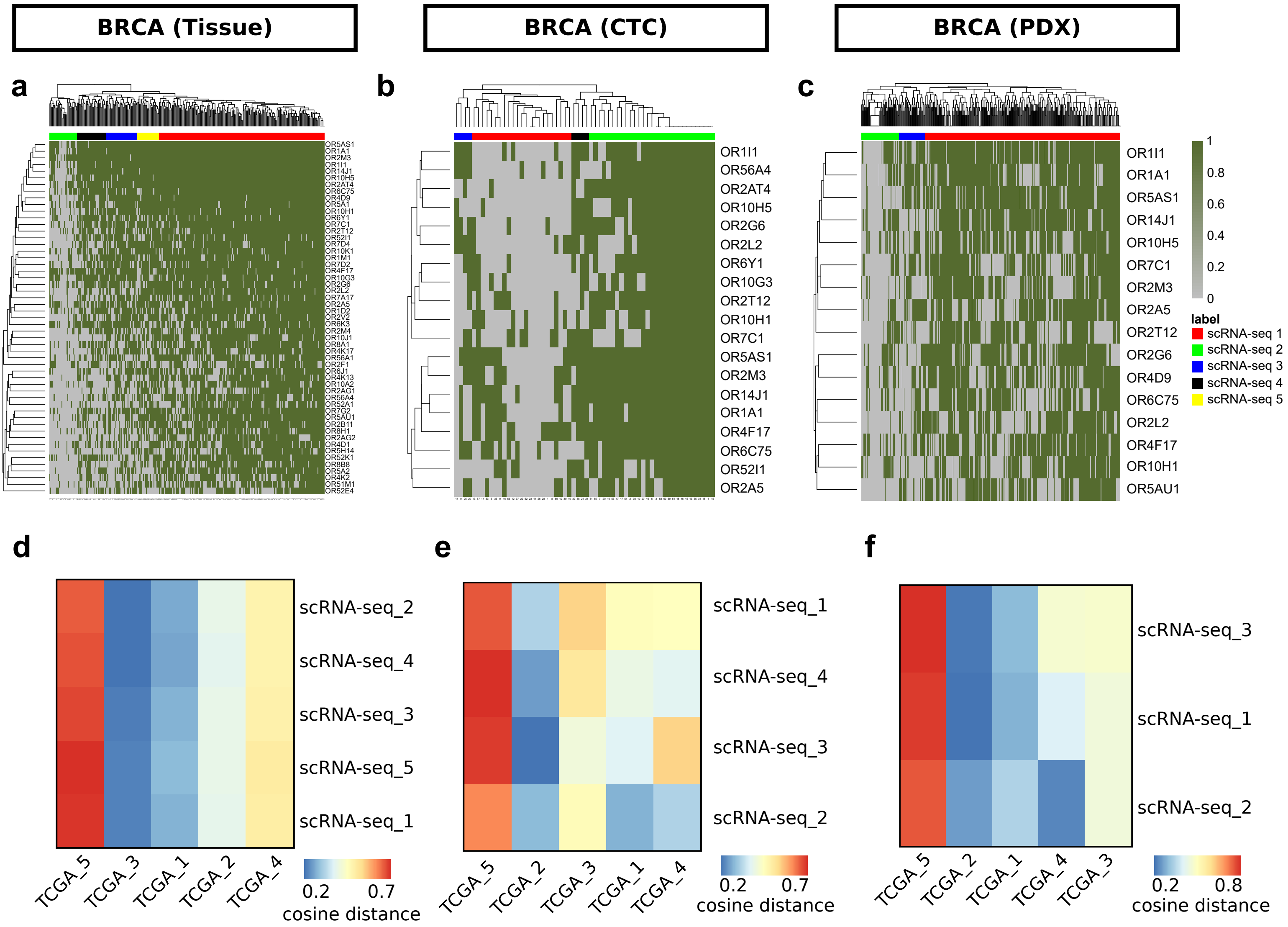


**Supplementary Figure 5**

**Supplementary Figure 6: Genomic alterations in breast carcinoma-associated OR genes.**

**(a-c)** Comparison of the tumor stages between two prominent clusters representing TCGA patient cohorts with distinct survival. Classification of the patients in distinct groups was performed using indicated single-cell dataset derived OR-centric signatures.

**(d-e)** Percentage bar graph representing the genomic alterations in breast carcinoma-associated ORs genes in the indicated datasets.

**(f)** Box plot representing the relative expression and genomic alteration in the representative breast carcinoma-associated ORs.

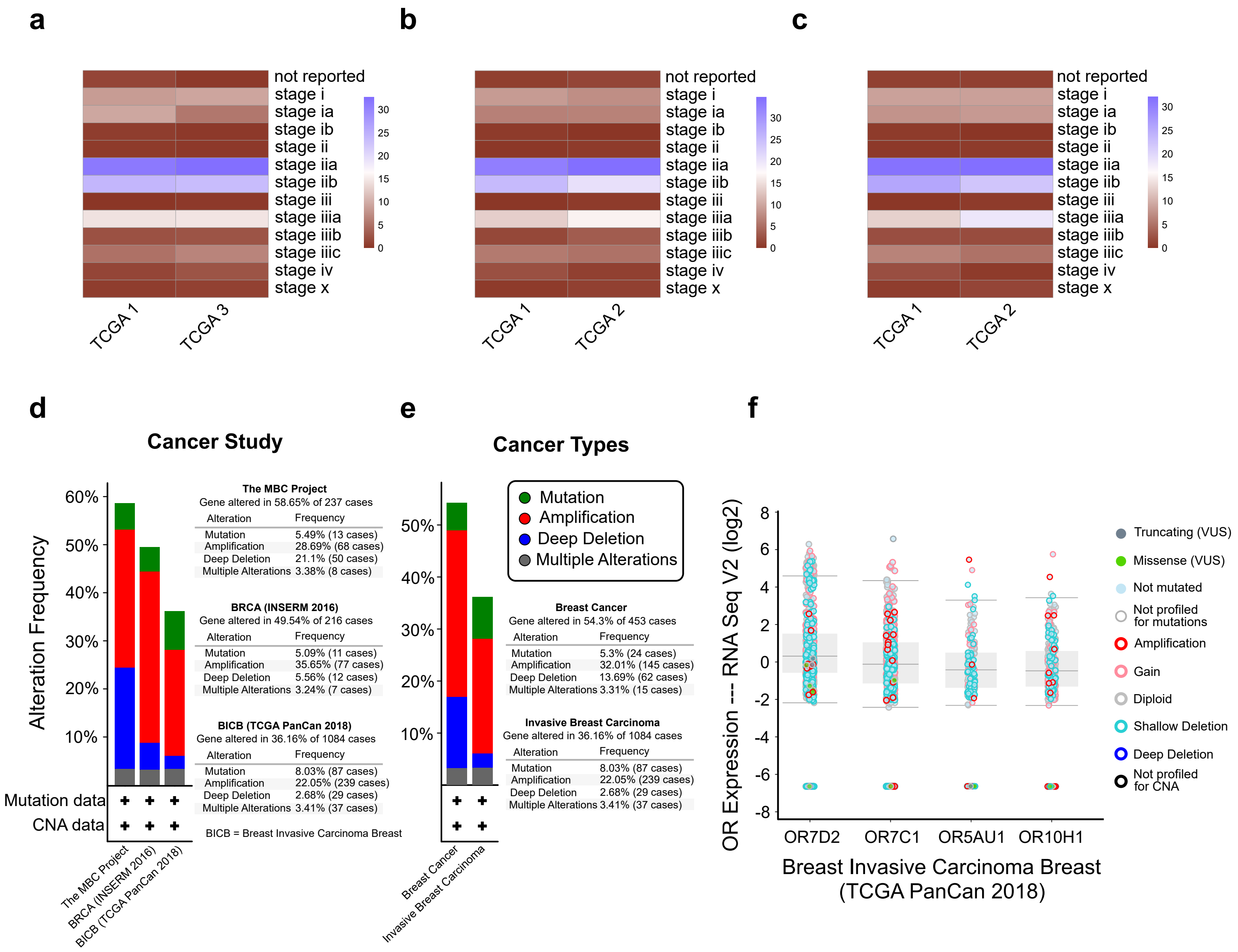


**Supplementary Figure 6**
